# *Trichoderma harzianum*- and Methyl Jasmonate-Induced Resistance to *Bipolaris sorokiniana* Through Enhanced Phenylpropanoid Activities in Bread Wheat (*Triticum aestivum* L.)

**DOI:** 10.3389/fmicb.2019.01697

**Published:** 2019-07-31

**Authors:** Udai B. Singh, Deepti Malviya, Shailendra Singh, Manoj Kumar, Pramod K. Sahu, H. V. Singh, Sunil Kumar, Manish Roy, Mohd. Imran, Jai P. Rai, A. K. Sharma, A. K. Saxena

**Affiliations:** ^1^Plant-Microbe Interaction and Rhizosphere Biology Lab, ICAR-National Bureau of Agriculturally Important Microorganisms, Mau, India; ^2^Department of Bioscience, Faculty of Applied Science, Integral University, Lucknow, India; ^3^Department of Mycology and Plant Pathology (Krishi Vigyan Kendra), Institute of Agricultural Sciences, Banaras Hindu University, Varanasi, India

**Keywords:** MeJA, Trichoderma harzianum, Bipolaris sorokiniana, hemi-biotroph, ISR, spot blotch disease

## Abstract

The aim of the present study was to evaluate the impact of *Trichoderma harzianum* UBSTH-501- and methyl jasmonate-induced systemic resistance and their integration on the spot blotch pathogen, *Bipolaris sorokiniana* through enhanced phenylpropanoid activities in bread wheat (*Triticum aestivum* L.). It was found that the application of MeJA (>100 mg L^–1^) inhibits the germination of *B. sorokiniana* spores under controlled laboratory conditions. To assess the effect of MeJA (150 mg L^–1^) in combination with the biocontrol agent *T. harzianum* UBSTH-501 *in vivo*, a green house experiment was conducted. For this, biocontrol agent *T. harzianum* UBSTH-501 was applied as seed treatment, whereas MeJA (150 mg L^–1^) was applied 5 days prior to pathogen inoculation. Results indicated that application of MeJA (150 mg L^–1^) did not affect the root colonization of wheat by *T. harzianum* UBSTH-501 in the rhizosphere. The combined application of *T. harzianum* UBSTH-501 and MeJA also enhanced indole acetic acid production in the rhizosphere (4.92 μg g^–1^ of soil) which in turn helps in plant growth and development. Further, the combined application found to enhance the activities of defense related enzymes *viz.* catalase (5.92 EU min^–1^ g^–1^ fresh wt.), ascorbate peroxidase [μmol ascorbate oxidized (mg prot)^–1^ min^–1^], phenylalanine ammonia lyase (102.25 μmol cinnamic acid h^–1^ mg^–1^ fresh wt.) and peroxidase (6.95 Unit mg^–1^ min^–1^ fresh wt.) significantly in the plants under treatment which was further confirmed by assessing the transcript level of *PAL* and *peroxidase* genes using semi-quantitative PCR approach. The results showed manifold increase in salicylic acid (SA) along with enhanced accumulation of total free phenolics, ferulic acid, caffeic acid, coumaric acid, and chlorogenic acid in the leaves of the plants treated with the biocontrol agent alone or in combination with MeJA. A significant decrease in the disease severity (17.46%) and area under disease progress curve (630.32) were also observed in the treatments with biocontrol agent and MeJA in combination as compared to *B. sorokiniana* alone treated plant (56.95% and 945.50, respectively). Up-regulation of phenylpropanoid cascades in response to exogenous application of MeJA and the biocontrol agent was observed. It was depicted from the results that PAL is the primary route for lignin production in wheat which reduces cell wall disruption and tissue disintegration and increases suberization and lignification of the plant cell as seen by Scanning Electron microphotographs. These results clearly indicated that exogenous application of MeJA with *T. harzianum* inducing JA- and/or SA-dependent defense signaling after pathogen challenge may increase the resistance to spot blotch by stimulating enzymatic activities and the accumulation of phenolic compounds in a cooperative manner. This study apparently provides the evidence of biochemical cross-talk and physiological responses in wheat following MeJA and biocontrol agent treatment during the bio-trophic infection.

## Introduction

Wheat (*Triticum aestivum* L.) is one of the most important cereal crops grown throughout the world and is the second staple food of South Asian countries including India. It has great nutritional as well as commercial importance in terms of its special functional composition and consumption demand. It ranks first in terms of the source of protein and second in terms of the source of calories in low- and middle-income countries like India, Nepal, Pakistan, Bangladesh, etc. (Joshi and Chand, 2011). However, the wheat crop suffers from a number of biotic and abiotic stress factors which cause severe yield losses every year (Joshi et al., 2007). The biotic factors include insect-pests, pathogens and others including both vertebrates and invertebrates. Among biotic stresses in wheat cultivation, the fungal pathogen, *Bipolaris sorokiniana* (Sacc.) Shoemaker (teleomorph *Cochliobolus sativus*) is of prime importance. It is an ascomycete with quite a wide host range in the Poaceae family and causes spot blotch disease in wheat. It is the most serious disease of wheat in India and probably ranks close to leaf rust in terms of destructiveness (Joshi and Chand, 2011; Singh et al., 2016a). The average yield losses due to spot blotch in wheat have been reported to be as high as 15.5% (Dubin and Ginkel, 1991) to 17% (Saari, 1998).

Management of spot blotch in wheat is largely dependent on the use of resistant cultivars and toxic chemical fungicides. Use of resistant cultivars is an important approach conferring sustainability to the agro-ecosystem but the breakdown of resistance over a period of time has still remained a great concern and it seems to be the major limitation of this approach (Singh G. et al., 2014; Singh et al., 2015, 2016a, 2019). Further, the non-target negative impact of plant protection chemicals, including fungicides, on both the environment and human health has obligated researchers to explore alternative measures for management of this disease. Among the more recent strategies, disease resistance induced by environment-friendly elicitors has emerged as a promising supplement in the approaches to crop protection (Schirra et al., 2011; Singh et al., 2015; Wang et al., 2015). Some of the phytohormones like jasmonic acid (JA), salicylic acid (SA), and ethylene (ET) act as signaling molecules that are essential for the regulation of plant defense responses to pathogenic stresses. Among these, JA plays an important role in the regulation of cellular immune responses in plants (Pieterse et al., 2012; Król et al., 2015; Sahu et al., 2016). Induction of the JA in response to pathogen(s) and herbivore(s) attack leads to a cascade of complex physio-biochemical changes within the host plant that contributes to the development of resistance/tolerance against wide range of invaders (Creelman and Mullet, 1997; Kaplan et al., 2008). It was also reported that JA activates expression of different genes encoding antifungal proteins such as thionin (Becker and Apel, 1992), osmotin (Xu et al., 1994), plant defencin (*Pdf 1.1* and *Pdf 1.2*) (Penninckx et al., 1996), and the ribosome-inactivating protein (*RIP* 60) (Chaudhry et al., 1994). JA modulates the expression of Proline-rich cell wall proteins (*PRPs*) in the plant cell that contributes to the synthesis of barriers which restrict the entry of pathogens (Creelman et al., 1992) and induces the genes involved in phytoalexin biosynthesis such as *chitinase*, *Phenylalanine ammonia lyase (PAL)* (Creelman et al., 1992; Choi et al., 1994) and phenolics (polyphenol oxidase). The key role of JA has been studied in model system *Arabidopsis* (Pieterse et al., 2012, 2014), whereas its role in elicitation of defense responses in wheat-spot blotch pathosystem still remains unexplored.

Plants have well-developed signaling mechanisms (Singh et al., 2003, Singh A. et al., 2013; Harman et al., 2004). They must make out the identities of the microbes in the vicinity and accordingly activate their respective mechanisms either to attract the desired microbial species or to keep the unwanted ones away. Perception and identification of pathogens by a host plant are, therefore, the key points in the activation of effective and rapid plant defense responses (de León and Montesano, 2013; Singh A. et al., 2014). It is mainly because of the innate ability of plants to recognize potential microbial invaders and modulate/re-program their defense system in accordance. However, many of the current insights have suggested that root-associated mutualistic rhizosphere microorganisms play an important role in the induction of systemic resistance in a wide range of crop species (Ciarkowska et al., 2016; Singh et al., 2016a; Gu et al., 2017; Mishra et al., 2018). Furthermore, JA may also help to regulate mutualist interactions between the plants and associated mutualistic microbes in both the above and belowground, but its role in the interaction process is less explored (Kiers et al., 2010). Some reports have indicated both positive and negative effects on mycorrhizal colonization when JA and MeJA applied exogenously to plant leaves. Kiers et al. (2010) suggested the concentration-dependent effects of jasmonates on mycorrhizal colonization. In general, mycorrhizal colonization was stimulated at low jasmonate concentrations and reduced at higher ones. As far as plant microbial interactions are concerned in a crop ecosystem, exogenous application of MeJA might have its own implications and it needs to be worked out through exhaustive investigations. *Trichoderma* is one of the microorganisms of great significance to crop cultivation and effects of exogenous application of MeJA on root colonization by *Trichoderma harzianum* still remains less explored.

*Trichoderma harzianum* is an avirulent plant symbiont and being ubiquitous in nature its presence has been recorded from across a number and variety of habitats. More so, it is known to be highly interactive in the plant root, soil and foliar environments (Harman, 2011; Sarma et al., 2015). Further, *T. harzianum* is a well-known bio-agent being effective against a broad spectrum of soilborne plant pathogenic fungi, bacteria and nematodes in a number of crop species (Harman et al., 2004; De Palma et al., 2016, 2019; Singh et al., 2016a,b, 2017; De Palma et al., 2019). It can colonize plant roots, both externally and internally (De Palma et al., 2019) and thus, is effective not only against an array of phytopathogenic microorganisms but also in improving the performance of the plant by and large. Moreover, *T. harzianum* grows chemotropically toward the roots of most of the cultivated plant species and stimulates the germination of propagules (be they active or dormant) of several plant pathogens in vicinity and finally inhibiting their growth or killing these propagules (Whipps, 1997; Vinale et al., 2008). It produces a diverse nature of metabolites such as xylanase and peptaibols that interfere with the plant directly or indirectly, leading to the induction of several defense-related cascades and innate immune responses against a wide range of invaders (Philippot et al., 2013; Shah and Zeier, 2013; Sarma et al., 2015; Pascale et al., 2017). More specifically, salicylate (SA) and jasmonate/ethylene (JA/ET)-signaling are activated in the plants immediately after *Trichoderma* invasion elicited JA/ET-mediated induced systemic resistance (Shoresh et al., 2010) and/or SA-mediated systemic acquired resistance (SAR) response, similar to that invoked by necrotrophic pathogens (Segarra et al., 2007; Contreras-Cornejo et al., 2011; Salas-Marina et al., 2011; Yoshioka et al., 2012). It was reported that *Trichoderma* spp. produce well-characterized elicitor Sm1/Epl1, small cysteine-rich hydrophobin-like protein of the cerato-platanin (CP) family that elicited ISR in maize (Djonovic et al., 2006, 2007; Seidl et al., 2006; Ahmad et al., 2016). Further, *Trichoderma* spp. may also affect the plant responses by activating its basic immunity or the microbe-associated molecular patterns (MAMPs)-triggered immunity (MTI), as well as reducing the effector-triggered susceptibility (ETS) and increasing the effector-triggered immunity (ETI) in the plant system (Harman et al., 2004; Lorito et al., 2010; Mukherjee et al., 2013; Sharma et al., 2017).

Furthermore, the synergistic effect of exogenous application of signaling molecule SA and MeJA with *T. harzianum* (Zehra et al., 2017a,b) and arbuscular mycorrhizal fungi (El-Khallal, 2007a,b) have been studied in tomato plants against Fusarium wilt disease. However, only a few studies to our knowledge have added MeJA with the *T. harzianum* to control wheat diseases. In view of the importance of spot blotch disease and role of JA and *T. harzianum* in the management of plant diseases, the present study was taken with the objective to explore the impact of root associated mutualistic plant symbiont, *T. harzianum*, methyl jasmonate and their combination in the induction of systemic resistance to hemi-biotroph *B. sorokiniana* through enhanced phenylpropanoid activities and other biochemical pathways in bread wheat.

## Materials and Methods

### Culture Medium and Reagents

Dehydrated potato dextrose agar (PDA) medium and potato dextrose broth (PDB) from HiMedia, India was used to grow both the fungal species, *viz.* the pathogen, *Bipolaris sorokiniana* UBS-101 and the biocontrol agent, *Trichoderma harzianum* UBSTH-501. Analytical grade chemical reagents and standards were procured from E. Merck, India. Methyl jasmonate (MeJA) was purchased from Sigma-Aldrich, India.

### Fungal Pathogen and Biocontrol Agent

Fungal pathogen *B. sorokiniana* UBS-101 and biocontrol agent *T. harzianum* UBSTH-501 were obtained from Plant–Microbe Interaction and Rhizosphere Biology Lab, ICAR-National Bureau of Agriculturally Important Microorganisms (ICAR-NBAIM), Kushmaur, Maunath Bhanjan, India. *B. sorokiniana* UBS-101 and *T. harzianum* UBSTH-501 were grown on PDA medium at 25 ± 2°C for 7 days and stored at 4°C till further use.

For liquid culture, *B. sorokiniana* UBS-101 was grown on PDB with incubation at 25 ± 2°C for 10 days. The colony forming unit (CFU) count of *B. sorokiniana* UBS-101 was 1.25 × 10^6^ ml^–1^ at the time of application. Spore suspension (2.5 ml plant^–1^) along with Tween 20 (50 μl) was applied on the plants to initiate disease under glasshouse conditions. However, talc-based bioformulation of *T. harzianum* UBSTH-501, named as Green Fungicide, was prepared as per the methods described by Singh et al. (2016b). The CFU count of the *T. harzianum* UBSTH-501 was done by serial decimal dilution on PDA. It was 1.32 × 10^6^ cfu g^–1^ in the talc-based formulation.

### Effects of MeJA on Spore Germination of *B. sorokiniana*

Effect of different concentrations of MeJA (50, 100, 150, and 200 μg ml^–1^) on spore germination of *B. sorokiniana* was assessed under *in vitro* conditions. One hundred spores of *B. sorokiniana* grown on PDB were placed into each concentration of MeJA (20 μL), incubated for 36 h and plated on water agar (1.5%). Thereafter, germinated spores were counted and spore germination (%) and inhibition of spore germination (%) were calculated using the following formulae (Chandel and Pimpalgaonkar, 2014):

$$\%\;\mathrm{Spore Germination}\;(\mathrm{SG})=\frac{\mathrm{Number of spores germinated}}{\mathrm{Total number of spores examined}}\times 100$$

$$\mathrm{Inhibition of spore germination}\;(\%)=\frac{\mathrm{C–T}}{C}\times 100$$

Where C = Number of germinated spores in control and T = Number of germinated spores in the treated one.

### Effects of MeJA on *T. harzianum* Root Colonization of Wheat

Effect of MeJA on root colonization of *T. harzianum* UBSTH-501 was studied in the wheat plants as per the methods described by Brotman et al. (2013) with slight modifications. Briefly, *T. harzianum* UBSTH-501 bioprimed seeds (wheat *cv.* HUW-234) were sown in pots containing sterile sand-soil-vermiculite mixture (1:1:1, w/w). Two plants in each pot were maintained and these plants were sprayed with MeJA (150 mg L^–1^) at a rate of 2.5 ml per plant at 20th day of sowing. Plants were uprooted at 30, 45, 60, 75, 90, and 120 days after sowing. Roots were detached and extensively washed under running tap water. Such washed roots were surface sterilized with 1% sodium hypochlorite for 1 min followed by triple washing with sterilized distilled water. The root samples were excised into small pieces of 2–3 cm, weighed, and homogenized in 25 ml of sterilized distilled water. Serial decimal dilutions were plated for cfu counts on *Trichoderma* selective medium and incubated at 25 ± 2°C (Vargas et al., 2009).

### Experimental Set-Up

The most promising strain, *T. harzianum*, UBSTH-501, selected from the preliminary *in vitro* study was assayed *in vivo* against *B. sorokiniana* UBS-101 infection in susceptible wheat cultivar (HUW-234). Untreated plants were used as negative controls, whereas plants treated solely with *B. sorokiniana* UBS-101 were kept as positive control. The experimental design comprised of five different treatments, *viz.* (i) plants treated only with the test pathogen *B. sorokiniana* UBS-101 (being positive control); (ii) plants treated with methyl-jasmonate (MeJA) + *B. sorokiniana* UBS-101; (iii) plants treated with antagonist *T. harzianum* UBSTH-501 + *B. sorokiniana* UBS-101; (iv) plants treated with antagonist *T. harzianum* UBSTH-501 + and MeJA + *B. sorokiniana* UBS-101; and (v) untreated plants (being the negative control).

*Trichoderma harzianum* UBSTH-501 was used as talc-based powdered formulations (named as Green Fungicide). Further, Tricho-AD, a mixture of adjuvant (Gum Acasia 0.01%) and additives (chitoson 1%, PVP 0.01% and Trehalose 0.01%) was worked out to be the best suited concentration for enhanced performance of the *Trichoderma* strain under study for seed treatment. Wheat seeds were bio-primed with *T. harzianum* UBSTH-501 formulation (at 5 g kg^–1^ of seed suspended in 10 ml Tricho-AD and 40 ml of water), air-dried in shade for 30 min, incubated for 6 h at ambient temperature and sown in the pots.

The spore suspension of *B. sorokiniana* containing 1.25 × 10^6^ ml^–1^ CFU was applied to initiate the disease at 45 days of sowing (Singh et al., 2016a). However, MeJA (150 mg L^–1^) was applied (at 2.5 ml plant^–1^) 5 days prior to the pathogen inoculation. Seeds treated with sterile talc powder (neither antagonist, nor the test phytopathogen) served as control. The moisture content in the pots was kept at field capacity (60%) by sprinkling sterilized water every alternate day. The growing conditions were: average temperature 22–25°C and relative humidity of 70–75% with a photoperiod of 11/13 h.

### IAA and Biochemical Analysis

Quantitative estimation of IAA in the rhizosphere soil was done as per the methods described by Thimmaiah (2012) after 30 days of pathogen inoculation. Briefly, rhizosphere soil was digested into distilled methanol (20 mL), filtered through G4 glass filter twice by re-adding distilled methanol (20 mL) and finally evaporating the methanol in a rotary evaporator at 30°C. Further, 10 mL of cold 0.5M K_2_HPO_4_ was added to the aqueous residue. The mixture was then, transferred to a separating funnel containing 10 mL light petroleum ether and shaken vigorously. This step was repeated with 10 mL diethyl ether and IAA extracted with 10 mL diethyl ether. To each flask containing IAA in diethyl ether 0.2 mL of ice-cold trifluroacetic acid- acetic anhydride was added and mixed properly. The reaction was stopped by adding 3 mL water and reading was recorded as excitation at 440 nm and emission at 490 nm.

Total phenolics in the plant leaves were measured as per the methods described by Sadasivam and Manickam (1996). Briefly, plant leaves were sampled (1.0 g), ground with 10 mL of 80% ethanol and centrifuged at 10000 rpm for 20 min. This step was repeated with 5 mL of 80% ethanol. The supernatant was evaporated in a rotary evaporator and 5 mL of distilled water was added to the aqueous residue. Further, different aliquots (0.2–2.0 mL) were taken into separate test tubes and the volume was adjusted to 3 mL by adding distilled water. Thereafter, Folin–Ciocalteau reagent (0.5 mL) was added into each test tube. After 3 min. Two mL of 20% Na_2_CO_3_ solution was added, mixed thoroughly and the tubes were placed in boiling water for 1 min. Finally, the absorbance was measured at 650 nm against a reagent blank.

Salicylic acid was extracted and measured as per the methods of Verberne et al. (2002). In short, fresh leaves (0.5 g) were sampled, homogenized in liquid nitrogen and transferred to a 1.5 mL Eppendorf tube. An aliquot (1 mL) of 90% methanol, 2.5 μL of the internal control compound 3,4-DHBA (10 μg/μL), and pure SA were added to the homogenate and rest of the steps given by Verberne et al. (2002) were followed. After removal from the concentrator, 600 μL of the HPLC eluent was added to each tube and the sample was analyzed for its contents accordingly. Further, individual phenolics such as caffeic acid, ferulic acid, *p*-coumaric acid, and chlorogenic acid were measured at 0, 12, 24, 36, 48, and 60 h after pathogen inoculation (hapi). The individual phenolics were extracted by following the steps given by Li et al. (2008). HPLC analysis was carried out using Shimadzu equipped with a photodiode array detector and C18 column. The wavelength used for quantification of phenolic acids was 280 nm. The flow rate of the mobile phase was 1.0 mL/min, and the injection volume was 20 μL.

For quantitative estimation of catalases, 1.0 g leaf tissue was homogenized in 150 M phosphate buffer (20 mL) at 4°C and diluted (1:10) by adding distilled water following the methods described by Sadasivam and Manickam (1996). The absorbance was taken at 240 nm and the catalase activity was calculated as unit g^–1^ leaf tissue. Similarly, activity of ascorbate peroxidase, phenylalanine ammonia lyase (PAL) and peroxidase activity was measured spectrophotometrically as per the methods described by Sadasivam and Manickam (1996) with slight modifications (Singh et al., 2016b) at 30 days after pathogen inoculation (dapi).

### RNA Isolation and Gene Expression Analysis

For gene expression analysis, a semi-quantitative method was used. Total RNA was extracted using RNA isolation kit (Agilent, India) following the manufacturer’s protocol from the wheat leaves taken from different treatments at 7 dapi. First-strand cDNA synthesis was performed using 1 μg of RNA primed with oligodT using cDNA Synthesis Kit (Bio-Rad, India) according to the manufacturer’s instructions/protocols. The gene (*Inducible PAL* and *peroxidase*) expression was analyzed using gene-specific primers (Table 1). PCRs of 20 μl with 1.2 μl of template cDNA were performed with 3U Taq DNA Polymerase (Bangalore GeNei, India). Actin was taken as control. Thermocycling was performed using Thermal Cycler (PaqStar) with the following cycling conditions: 95°C for 4 min, 35 cycles of 94°C for 45 s, 50.7°C (*PAL*) or 53°C (*peroxidase*) or 55°C (*actin*) for 45 s; 72°C for 60 s, followed by final extension of 72°C for 10 min. The final product obtained with RT-PCR was separated by electrophoresis in 1.2% agarose gel in TAE buffer using gel electrophoresis apparatus (Bangalore GeNei, India) and visualization was done in gel documentation system (Bio-Rad, India).

**TABLE 1:** Primers used for semi-quantitative real-time reverse transcription PCR.

Target gene	RT-PCR-primers sequence	Product size (bp)
Actin	Actin 1-F-CGAAGCGACATACAATTCCA Actin 1-R-AATAGAGCCACCGATCCAGA	211
	Actin 2-F-CCAGCCATCTCATGTTGGTA Actin 2-R-AATAGAGCCACCGATCCAGA	250
	Actin 3-F-TGGTTCAGAAAGGTTCAGGTG Actin 3-R-GAAAGTGCTAAGAGAGGCCAAA	307
	Actin 4-F-CCAGCAATGTATGTCGCAAT Actin 4-R-AGTCCCCTTCACCGACTCTT	760
*Inducible* PAL	*PAL* 1-F-CCAATGTTCTGTCCGTCCTT *PAL* 1-R-TGATCTCACGCTCAATCGAC	309
	*PAL* 2-F-CCAATGTTCTGTCCGTCCTT *PAL* 2-R-GCCCTTGAAACCATAGTCCA	545
	*PAL* 3-F-CCAATGTTCTGTCCGTCCTT *PAL* 3-R-TCACCGCTGTCTTCATGTTC	783
*Class III* Peroxidase	*POX* 1-F-GGCATGGAACAAAACGCTAT *POX* 1-R-TGATACTCTTACGGCGACGA	162
	*POX* 2-F-GCAGAGTATGCTGCCAACCT *POX* 2-R-GCCACCGGTCTTCACTTCTA	282
	*POX* 3-F-GCGCATACACTCACCCCTAT *POX* 3-R-GTTCCGATGTTGGTCTCGTT	324
	*POX* 4-F-ATCTCGCACTGCAACTCCTT *POX* 4-R-GCCACCGGTCTTCACTTCTA	366
	*POX* 5-F-AAACCTCACCACCTTGTTCG *POX* 5-R-GTTCCGATGTTGGTCTCGTT	506

### Effects of Treatments on Spore Germination and Disease Dynamics

Effects of crude extract of wheat leaves extracted from plants pre-treated with *T. harzianum* UBSTH-501 and foliar spray of MeJA (150 mg L^–1^) on spore germination of *B. sorokiniana* UBS-101 were recorded as per the methods described by Sahu et al. (2016) with slight modifications. Briefly, after 10 days of pathogen inoculation, plant leaves were sampled from each treatment. Thereafter, leaves were washed under running tap water followed by thorough rinsing with distilled water. The excess water on the surface of the leaves was removed by absorbing it with blotter paper; ground and crude extract was separated by centrifugation and filtration (syringe filter, 0.22 μm). One hundred spores of *B. sorokiniana* were added separately into leaves extract obtained from each treatment for 36 h. Thereafter, these spores were plated on water agar and incubated for 36 h at 25 ± 2°C. The germinated spores were then counted and germination (%) was calculated using the following formula:

$$\mathrm{Germination}\;(\%)=\frac{\mathrm{Total number of spores germinated}}{\mathrm{Total number of spores plated}}\times 100$$

Inoculated plants were watched regularly for disease progress and disease severity (%) and area under disease progress curve (AUDPC) was calculated as per the methods described by Singh et al. (2016a).

### Effects of Treatments on Lignin Content in Plant Leaves

Lignin content was recorded in the plant leaves as described by Fredrik and Elisabeth (2011) with slight modifications (Yusuf et al., 2016). Briefly, leaves were sampled from each treatment at 30 dapi and dried at 50°C for 36 h until a constant weight was obtained. Thereafter, 100 mg dried leaf sample was digested in 1 ml 12M H_2_SO_4_, then 28 ml distilled water was added to it and the solution was incubated at 30 ± 0.5°C for 1 h with occasional stirring. Hydrolysis was done at 120 ± 5°C for 1 h and the solution was filtered while still hot and acid-soluble lignin present in the filtrate was estimated spectrophotometrically at 205 nm (UV-VIS Spectrophotometer, Shimadzu UV-2450).

### Histo-Pathological Study

For observation of lignin deposition, plant leaves were sampled at 30 dapi, transverse sections (30 μ) were cut in a microtome (Biogen) followed by their staining. Differential staining was carried out as per the methods described by Jensen (1962). Briefly, the sections were fixed in ethanol (gradients 10, 25, 50 75, 80, and 95% v/v), mounted on a glass slide in a solution of saturated aqueous phloroglucinol (prepared in 20% HCl) and observed under light microscope at 40X (Olympus BX 41, Japan). The positive lignin staining was indicated by red-violet color under compound light microscope.

Similarly, for observation of pathogen colonization, plant leaves were sampled at 30 dapi for scanning electron microscopic study. Samples were fixed in 2.5% glutaraldehyde and osmium tetraoxide solution (HiMedia, India). Leaves were cut into small pieces, dehydrated using gradient of ethyl alcohol (5, 10, 20, 50, 70, 90, 100%) and dried under vacuum. Further, gold coating (20 nm) was done before visualization. However, confocal scanning laser microscopy was done using 488 and 543 nm laser lines (Nikon Eclipse Confocal A1). Phloxin-B was used for staining the fungal pathogen. The Z-stake was captured to visualize the 3D image showing infection structure and mode of penetration in the leaf and visualized with a background filter of 488 nm.

Furthermore, to visualize the tissues disintegration and cell disruption, transverse sections (30 μ) were cut in a microtome (Biogen). These sections were further fixed in 2.5% glutaraldehyde and osmium tetraoxide solution and dehydrated (as mentioned above). The observations were taken using scanning electron microscope (HITACHI, S-3400N Model).

### Statistical Analyses

The laboratory experiments were laid out in completely randomized design in ten replications. Glasshouse experiments were laid out in complete randomized block design in ten replications and the data were subjected to analysis of variance and least significant difference (LSD) at *p ≤* 0.05 using statistical package for Social Sciences Version 16.0 program (SPSS Inc., 2007). Data were compared with Duncan’s multiple range test at *p ≤* 0.05. Graphs were prepared using statistical software Origin (Version 9) and Microsoft Office Excel (2010).

## Results

### Effects of MeJA on Spore Germination of *B. sorokiniana* UBS-101

**FIGURE 1:**
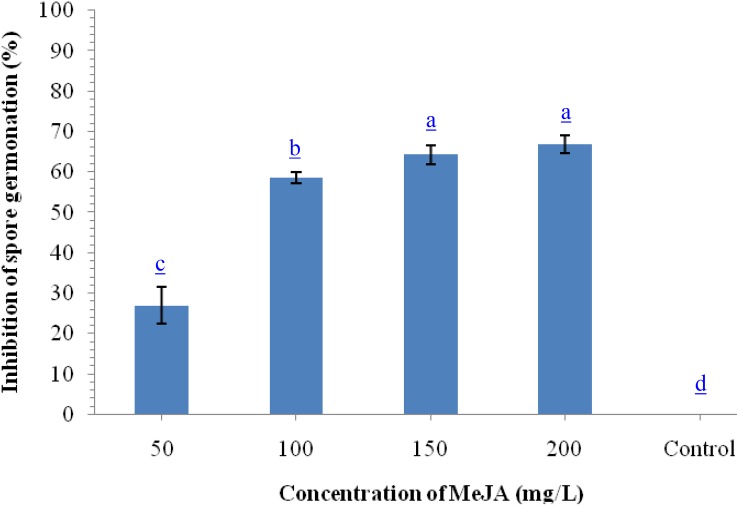
Effects of different concentration of methyl-jasmonate (MeJA) on inhibition of spore germination (%) of *B. sorokiniana* after 36 h under *in vitro* conditions. Data are mean (*n* = 5) and vertical bar lines represent standard error of mean, data with different letters show significant difference in column data in randomized block design test at *p* < 0.05 under Duncan’s multiple range test.

To monitor the antifungal properties of MeJA, we performed *in vitro* assay using different concentrations of MeJA against *B. sorokiniana* UBS-101. Results indicated that maximum inhibition of spore germination (67.98%) was recorded at 200 μg ml^–1^ concentration followed by 150 μg ml^–1^ (64.29%) and 100 μg ml^–1^ (62.68%), while, minimum inhibition was recorded on 50 μg ml^–1^ concentration (Figure 1).

### Effect of Biocontrol Agent and MeJA Application on Root Colonization by *T. harzianum*

**FIGURE 2:**
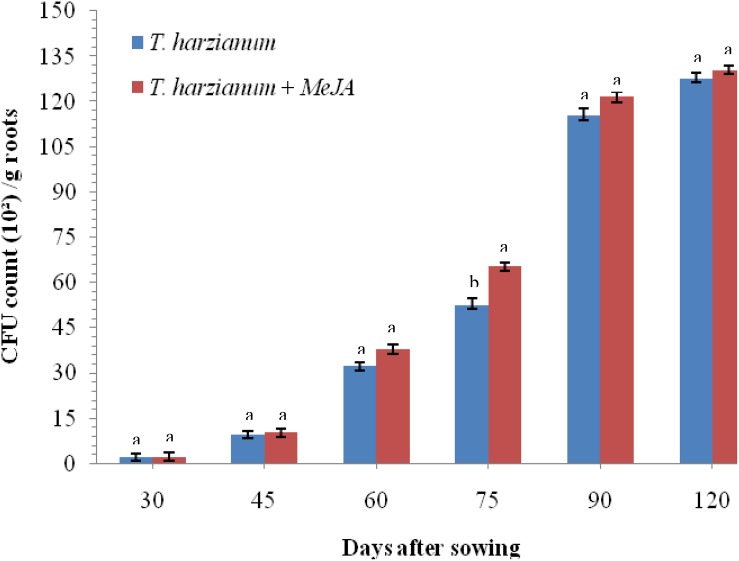
Effects of MeJA on root colonization of wheat by *Trichoderma harzianum* at 30, 45, 60, 75, 90, and 120 DAS under pathogenic stress conditions. Data are mean (*n* = 5) and vertical bar lines represent standard error of mean, data with different letters show significant difference in column data in randomized block design test at *p* < 0.05 under Duncan’s multiple range test.

To investigate whether application of MeJA is having any negative effects on root colonization by *T. harzianum* UBSTH-501, an experiment was conducted under glasshouse conditions. Results clearly indicate that treatment with MeJA (150 mg L^–1^) did not result in any reduction in the cfu count as well as root colonization ability of *T. harzianum* UBSTH-501 compared to that in plants treated with *T. harzianum* UBSTH-501 alone after 30, 45, 60, 75, 90, and 120 days of sowing (Figure 2).

### Effect of *T. harzianum* Root Colonization and MeJA Application on IAA Production

**FIGURE 3:**
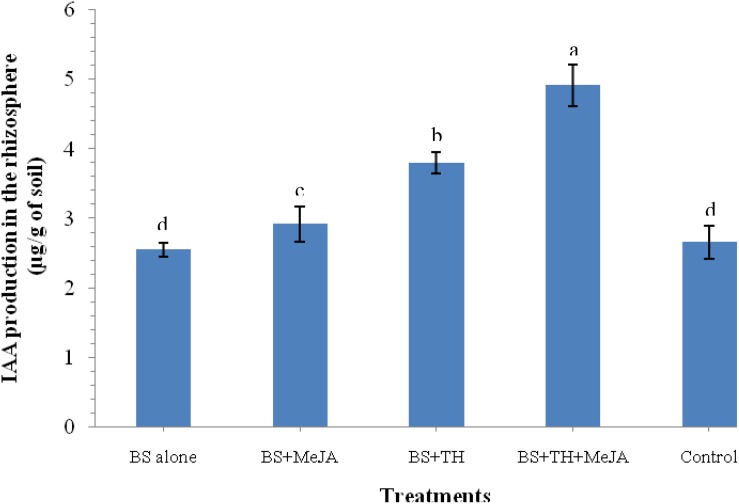
Effects of biocontrol agent, *Trichoderma harzianum* UBSTH-501 and methyl-jasmonate (MeJA) application on IAA production in the wheat rhizosphere at 30 dapi, treatments: 1 – *B. sorokiniana* (alone), 2 – *B. sorokiniana* + methyl-jasmonate (MeJA), 3 – *B. sorokiniana* + *T. harzianum* UBSTH-501, 4 – *B. sorokiniana* + *T. harzianum* UBSTH-501 + MeJA, and 5 –control (untreated). Data are mean (*n* = 5) and vertical bar lines represent standard error of mean, data with different letters show significant difference in column data in randomized block design test at *p* < 0.05 under Duncan’s multiple range test.

An increased level of IAA in the rhizosphere soil that contributes in general plant growth promotion under pathogenic stress was pointed out by the results of the biochemical analysis. Results showed that plants treated with *T. harzianum* UBSTH-501 and MeJA have a positive effect on the level of IAA in the rhizosphere soil. A significantly higher level of IAA was recorded in the plant rhizosphere treated with *T. harzianum* UBSTH-501 and MeJA in combination as compared to those treated with *T. harzianum* UBSTH-501 or MeJA alone under pathogenic stress conditions (Figure 3).

The effects on enhancement of IAA were also evident from increased plant growth parameters. Plants treated with *T. harzianum* UBSTH-501 and MeJA in combination significantly (*P ≤* 0.05) promoted plant growth parameters such as plant height, number of tillers per plants and dry biomass in the pots with and without the test fungal pathogen, *B. sorokiniana* UBS-101 compared to untreated control plants at 30, 60, 90, and 120 days of sowing (Table 2).

**TABLE 2:** Effect of *T. harzianum* UBSTH-501 and MeJA (150 mg L^–1^) treatment on different growth parameters of wheat plant grown in pots under pathogen challenged conditions.

**Treatments**	**Plant height (cm)**				**Number of tillers plant ^–1^**				**Plant biomass on dry wt. basis (g)**			
	**30 DAS^†^**	**60 DAS**	**90 DAS**	**120 DAS**	**30 DAS**	**60 DAS**	**90 DAS**	**120 DAS**	**30 DAS**	**60 DAS**	**90 DAS**	**120 DAS**
**With fungal pathogen**												
Control (untreated)	16.25 ± 1.14^b^	34.66 ± 2.15^b^	62.50 ± 2.25^b^	76.04 ± 1.57^b^	2.25 ± 0.33^b^	4.05 ± 0.45^b^	4.90 ± 0.25^b^	4.92 ± 0.45^b^	1.35 ± 0.22^b^	3.20 ± 0.45^b^	4.95 ± 0.23^b^	5.75 ± 0.25^b^
*T. harzianum* and	25.33 ± 2.25^a^	52.50 ± 2.33^a^	79.65 ± 2.50^a^	82.50 ± 3.25^a^	3.50 ± 0.25^a^	6.33 ± 0.75^a^	6.80 ± 0.66^a^	6.85 ± 0.40^a^	1.82 ± 0.11^a^	4.10 ± 0.25^a^	5.75 ± 0.35^a^	8.20 ± 0.55^a^
MeJA treated												
**Without fungal pathogen**												
Control (untreated)	17.50 ± 1.02^b^	38.60 ± 2.25^b^	70.30 ± 2.50^b^	86.05 ± 1.57^b^	2.75 ± 0.55^b^	5.66 ± 0.36^b^	6.50 ± 0.25^b^	6.96 ± 0.66^b^	1.89 ± 0.33^b^	4.40 ± 0.50^b^	6.21 ± 0.33^b^	6.88 ± 0.45^b^
*T. harzianum* and	24.70 ± 2.01^a^	61.90 ± 2.36^a^	95.40 ± 3.50^a^	100.30 ± 2.65^a^	4.25 ± 0.22^a^	8.40 ± 1.02^a^	8.90 ± 0.66^a^	8.90 ± 0.75^a^	2.38 ± 0.25^a^	5.54 ± 0.15^a^	8.20 ± 0.25^a^	10.50 ± 0.75^a^
MeJA treated												

*^†^DAS represent days after sowing, data are mean ± SEM, values with in a column followed by the same letter are not significantly different at p < 0.05.*

### Effect of Biocontrol Agent and MeJA Application on Enzymatic Activities

**FIGURE 4:**
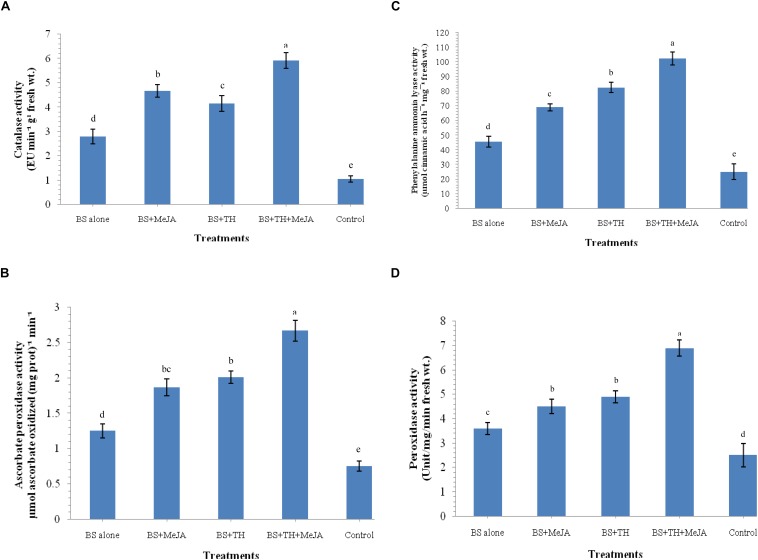
Activities of defense-related enzymes induced in wheat plants treated with *T. harzianum* and MeJA, **(A)** catalase, **(B)** ascorbate peroxidase, **(C)** phenylalanine ammonia lyase (PAL), **(D)** peroxidase at 30 dapi, treatments were: 1 – *B. sorokiniana* (alone), 2 – *B. sorokiniana* + methyl-jasmonate (MeJA), 3 – *B. sorokiniana* + *T. harzianum* UBSTH-501, 4 – *B. sorokiniana* + *T. harzianum* UBSTH-501 + MeJA, and 5 –control (untreated). Data are mean (*n* = 5) and vertical bar lines represent standard error of mean, data with different letters show significant difference in column data in randomized block design test at *p* < 0.05 under Duncan’s multiple range test.

The activation of defense-related enzymes was studied in the wheat plants treated with *T. harzianum* UBSTH-501 and MeJA (individually and in combination) and challenged with *B. sorokiniana* UBS-101 on 30 dapi. The activities of catalase, ascorbate peroxidase, PAL and peroxidase were significantly enhanced in the plants treated with *T. harzianum* UBSTH-501 and MeJA individually or in combination as compared to the positive control (*B. sorokiniana* UBS-101 inoculated) and negative control plants (untreated) at 30 dapi (Figures 4A–D). The maximum activity (2.12-fold) of catalase was found in the plant treated with both the agents in combination (5.92 EU min^–1^ g^–1^ fresh wt.) compared to *B. sorokiniana* UBS-101 inoculated plants (2.79 EU min^–1^ g^–1^ fresh wt.), whereas, this value was as high as 5.63-fold over the untreated control plants (1.05 EU min^–1^ g^–1^ fresh wt.) at 30 dapi (Figure 4A). However, no significant difference in catalase activity was recorded between the plants treated with *T. harzianum* UBSTH-501 alone and those treated with MeJA alone at 30 dapi (Figure 4A). Similarly, ascorbate peroxidase activity was found to be 2.13 and 3.56-fold in the plants treated with *T. harzianum* UBSTH-501 and MeJA in combination [2.67 μmol ascorbate oxidized (mg prot.)^–1^ min^–1^] compared to *B. sorokiniana* UBS-101 inoculated [1.25 μmol ascorbate oxidized (mg prot.)^–1^ min^–1^] and untreated control plants [0.75 μmol ascorbate oxidized (mg prot.)^–1^ min^–1^] at 30 dapi (Figure 4B). No significant differences in ascorbate peroxidase activity were detected between plants treated individually either with *T. harzianum* UBSTH-501 or with MeJA at 30 dapi (Figure 4B).

At 30 dapi, all the treatments and even the positive control showed increased activity of PAL as compared to the untreated (negative) control plants (Figure 4C). Treatment with *T. harzianum* UBSTH-501 and MeJA in combination under pathogen challenged showed an increment of PAL by 2.25 and 4.10-fold in plant leaves (102.25 μmol cinnamic acid h^–1^ mg^–1^ fresh wt.) as compared to pathogen *B. sorokiniana* UBS-101 inoculated or positive control (45.26 μmol cinnamic acid h^–1^ mg^–1^ fresh wt.) and untreated or negative control plants (24.92 μmol cinnamic acid h^–1^ mg^–1^ fresh wt.), respectively. However, a significant rise in PAL activity was recorded in plants treated even either with *T. harzianum* UBSTH-501 or with MeJA individually after 30 dapi (Figure 4C). Similar to other enzymes, manifold increase in the accumulation and activity of peroxidase was recorded in the plant leaves inoculated with *T. harzianum* UBSTH-501 and MeJA alone and in combination under pathogenic stress conditions as compared to untreated control plants at 30 dapi (Figure 4D). However, no significant difference in the peroxidase activity was recorded in the plants treated with *T. harzianum* UBSTH-501 and MeJA individually at 30 dapi under pathogenic stress (Figure 4D).

### Effect of Biocontrol Agent and MeJA Application on Phenolics and SA in Wheat

**FIGURE 5:**
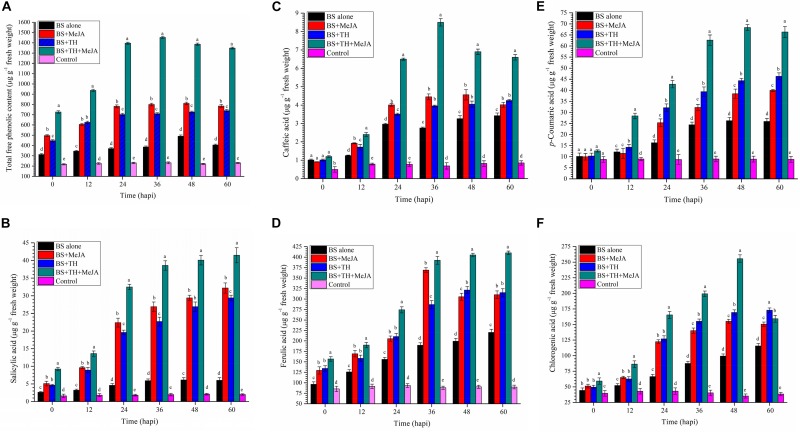
Activities of defense-related molecules induced and accumulated in wheat plants treated with *T. harzianum* and MeJA, **(A)** Total free phenolics, **(B)** SA, **(C)** caffeic acid, **(D)** ferulic acid, **(E)** *p*- coumaric acid, and **(F)** chlorogenic in wheat plants at 0, 12, 24, 36, 48, and 60 hapi, treatments: 1 – *B. sorokiniana* (alone), 2 – *B. sorokiniana* + methyl-jasmonate (MeJA), 3 – *B. sorokiniana* + *T. harzianum* UBSTH-501, 4 – *B. sorokiniana* + *T. harzianum* UBSTH-501 + MeJA, and 5 –control (untreated). Data are mean (*n* = 5) and vertical bar lines represent standard error of mean, data with different letters show significant difference in column data in randomized block design test at *p* < 0.05 under Duncan’s multiple range test.

Root colonization by *T. harzianum* UBSTH-501 coupled with MeJA application showed significant differences in terms of total phenolics; SA and individual phenolic content in the wheat plants under pathogenic stress as compared to *B. sorokiniana* UBS-101 alone inoculated (positive control) and untreated control/negative control plants (Figures 5A–F). Pathogenic stress caused a sharp increase in the total phenolic content at 36 hapi. Plants treated with *T. harzianum* and MeJA in combination, significantly (*P ≤* 0.05) enhanced expression and accumulation of total free phenolics in their leaves (1452.25 μg g^–1^ fresh wt.) as compared to other treatments at 36 hapi. After 36 hapi, a slight decrease was recorded in the total free phenolic content at 48 and 60 hapi in the plants treated with *T. harzianum* and MeJA in combination under pathogenic stress. No significant change in the phenolic content was found in untreated control plants at different time intervals (Figure 5A).

Results of the investigation further revealed that exogenous application of MeJA alone or in combination with the biocontrol agent, *T. harzianum* UBSTH-501 significantly increased the endogenous level of SA in wheat plants challenged with spot blotch pathogen, *B. sorokiniana* UBS-101 (41.50 μg g^–1^ fresh wt.). In pathogen challenged plants treated with both UBSTH-501 and MeJA, the analyses revealed an approximately 6.5- and 20.0-fold increase, respectively in leaf SA content when compared to the pathogen challenged (positive) and unchallenged (negative) controls (6.01 and 2.0 μg g^–1^ fresh wt., respectively) after 60 hapi. Interestingly, in the leaves of the plants treated with MeJA alone, SA content was significantly higher (32.25 μg g^–1^ fresh wt.) in comparison to those treated with *T. harzianum* UBSTH-501 alone (29.45 μg g^–1^ fresh wt.) under pathogenic stress conditions at 24, 36, and 60 hapi. However, under pathogenic stress a trend of leaf SA content of MeJA treated plants being higher or at par with leaf SA content in plants treated with *T. harzianum* was observed at all the time intervals (Figure 5B).

Similarly, the activation and accumulation of individual phenolics were studied in wheat plants treated with *T. harzianum* UBSTH-501 individually and in combination of MeJA under pathogenic stress of *B. sorokiniana* UBS-101. Results showed that maximum accumulation of caffeic acid was recorded in plants treated with *T. harzianum* UBSTH-501 combined with MeJA after 36 hapi (Figure 5C). However, no significant difference was recorded in the plants treated with *T. harzianum* UBSTH-501 and MeJA alone at this point of observation onward. However, the maximum increase in ferulic acid content was recorded in plants treated with *T. harzianum* UBSTH-501 and MeJA in combination under pathogenic stress after 60 hapi (Figure 5D). Similarly, *p*- coumaric acid and chlorogenic acid were observed to be maximum in plants treated with MeJA and *Trichoderma harzainum* under pathogenic stress after 48 hapi (Figures 5E,F, respectively). Additionally, the content of individual phenolics in the plant leaves of all the treatments without pathogen showed non-significant results compared to plants treated with pathogen alone or in a combination of biocontrol agent and MeJA at different time intervals (Figures 5C–F).

### Gene Expression Analysis

**FIGURE 6:**
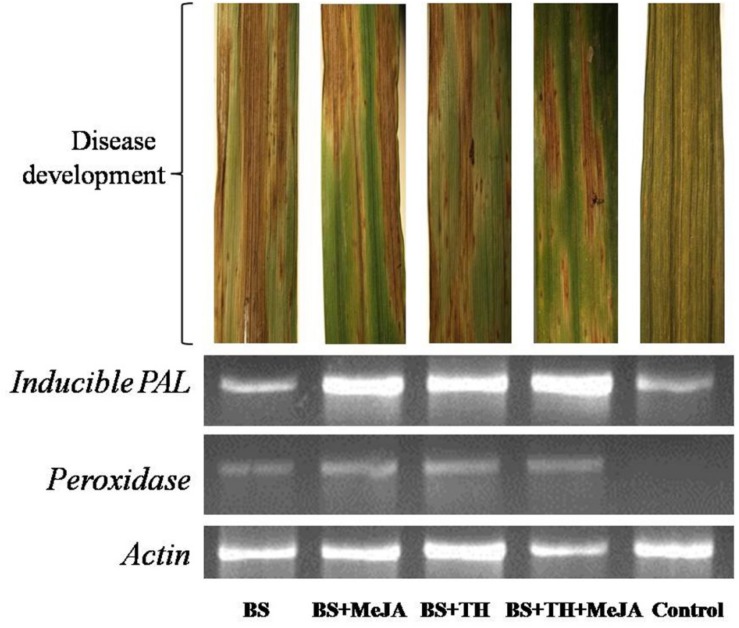
Effects of *T. harzianum* and MeJA application on PAL and peroxidase gene expression at 7 dapi, treatments were: 1 – *B. sorokiniana* (alone), 2 – *B. sorokiniana* + methyl-jasmonate (MeJA), 3 – *B. sorokiniana* + *T. harzianum* UBSTH-501, 4 – *B. sorokiniana* + *T. harzianum* UBSTH-501 + MeJA, and 5 –control (untreated).

Results showed that the application of *T. harzianum* UBSTH-501 and MeJA up-regulated phenylpropanoid cascades. Semi-quantitative gene expression analysis showed that *PAL* and *peroxidase* genes expressed significantly in the plants pre-treated with *T. harzianum* UBSTH-501 and MeJA either alone or in combination with each other as compared to both the controls (Figure 6). However, comparatively less gene expression was recorded in plants treated with the pathogen (alone) and untreated control plants.

### Effect of Biocontrol Agent and MeJA Application on the Tissue Disintegration and Cell Suberization

**FIGURE 7:**
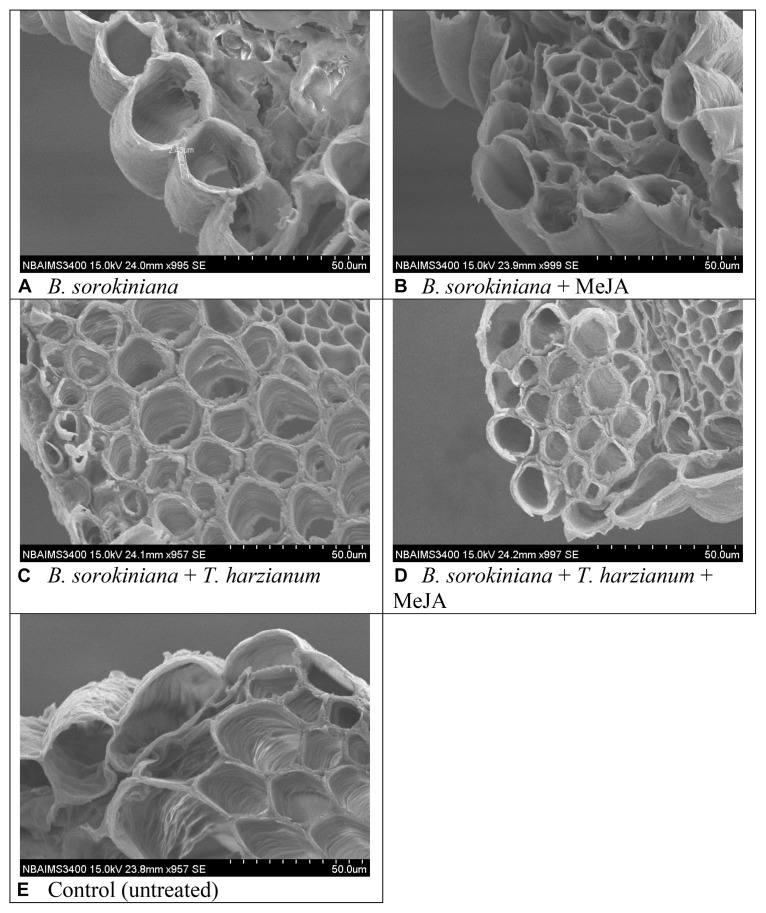
Effects of *T. harzianum* and MeJA application on tissue disintegration and cell suberization in the plant leaves at 30 dapi, treatments were: **(A–E)** 1 – *B. sorokiniana* (alone), 2 – *B. sorokiniana* + methyl-jasmonate (MeJA), 3 – *B. sorokiniana* + *T. harzianum* UBSTH-501, 4 – *B. sorokiniana* + *T. harzianum* UBSTH-501 + MeJA, and 5 –control (untreated).

Scanning electron microphotographs clearly showed that application of biocontrol agent and MeJA alone and in combination significantly reduced the cell wall disruption and tissue disintegration in the plant leaves (Figure 7). Maximum cell wall disruption and tissues disintegration were observed in the leaves inoculated with *B. sorokiniana* alone (positive control) as compared to other treatments (Figure 7A). Similarly, maximum cell suberization was in the plant leaves treated with *T. harzianum* UBSTH-501 and MeJA in combination (Figure 7D). It was clearly observed that intercellular spaces were completely packed in plant leaves treated with both the agents which might have restricted the test pathogen from further colonization and invasion of the deeper tissues (Figures 7B–D).

### Effect of Biocontrol Agent and MeJA Application on Lignin Content

**FIGURE 8:**
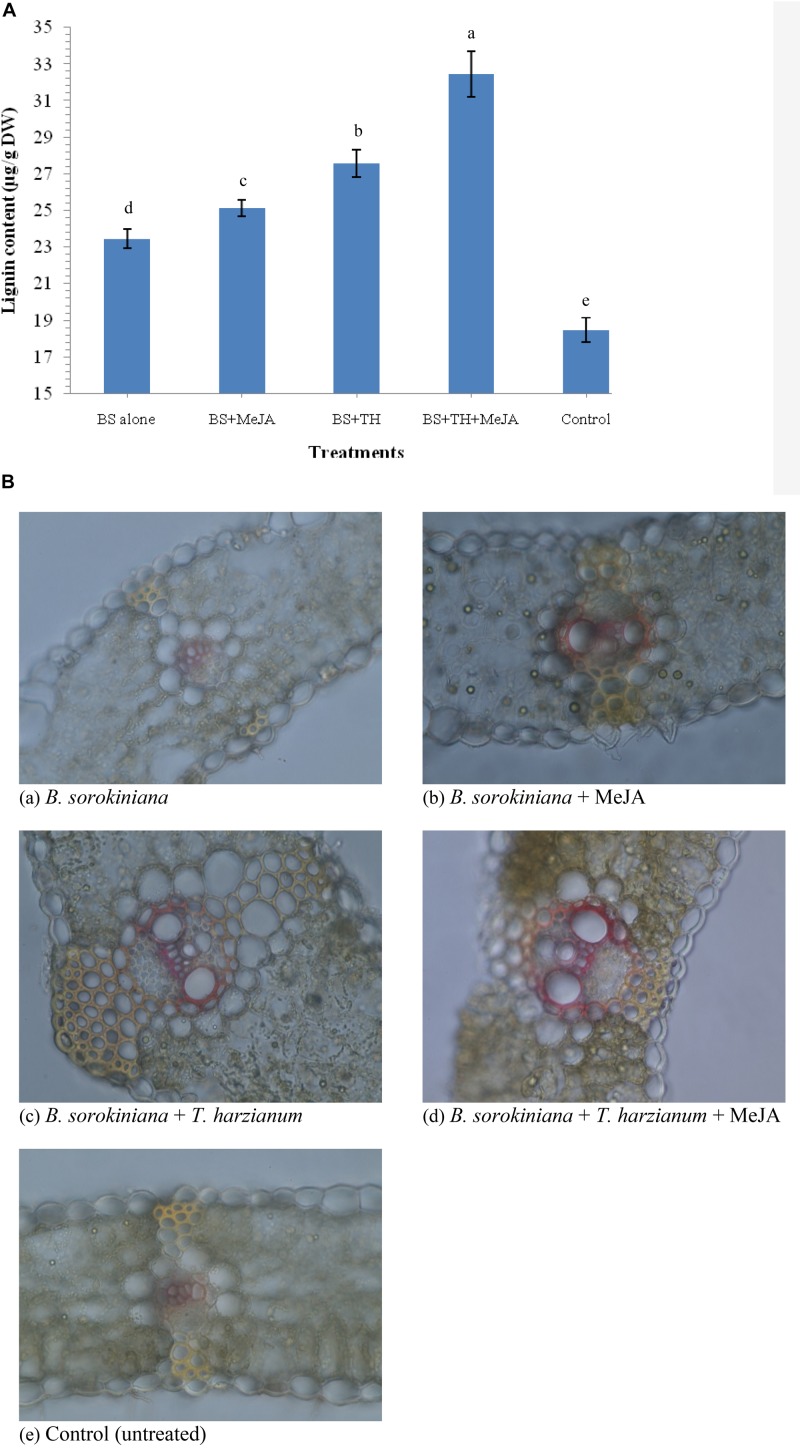
Effects of *T. harzianum* and MeJA application on **(A)** lignin content, and **(B)** histological deposition of lignin in the plant leaves at 30 dapi, treatments were: (a–e) 1 – *B. sorokiniana* (alone), 2 – *B. sorokiniana* + methyl-jasmonate (MeJA), 3 – *B. sorokiniana* + *T. harzianum* UBSTH-501, 4 – *B. sorokiniana* + *T. harzianum* UBSTH-501 + MeJA, and 5 –control (untreated). Data are mean (*n* = 5) and vertical bar lines represent standard error of mean, data with different letters show significant difference in column data in randomized block design test at *p* < 0.05 under Duncan’s multiple range test.

Application of biocontrol agent *T. harzianum* UBSTH-501 and MeJA individually or in combination induced the synthesis and accumulation of lignin in the leaf tissue under pathogenic stress. The quantitative estimation of lignin revealed that its content varied significantly in plants treated with biocontrol agent and MeJA individually or in combination when compared to plants treated with the test pathogen alone (positive control) and untreated/negative control plants at 30 dapi (Figure 8A). The maximum amount of lignin was recorded in the plant leaves treated with biocontrol agent and MeJA in combination (32.46 μg g^–1^ dry wt.) followed by those treated with biocontrol agent alone (27.56 μg g^–1^ dry wt.) and MeJA alone (25.15 μg g^–1^dry wt.). However, minimum amount of lignin was recorded in leaves of the untreated control plants (18.47 μg g^–1^ dry wt.) followed by plant leaves treated with the test fungal pathogen alone (23.46 μg g^–1^ dry wt.) at 30 dapi (Figure 8A).

Plants treated with *T. harzianum* UBSTH-501 and MeJA alone or in combination showed significant variations in lignin deposition. Histopathological study clearly indicated that significantly higher and uniform lignification was observed in the microphotographs of plant leaves treated with the *T. harzianum* UBSTH-501 and MeJA in combination followed by *T. harzianum* UBSTH-501 and MeJA individually treated plants as compared to those treated with pathogen alone (positive control) and untreated negative control plants (Figure 8B). Results revealed that maximum and uniform lignin deposition in vascular bundles and pericycle were observed in the plant leaves jointly treated with *T. harzianum* UBSTH-501 and MeJA under pathogenic stress condition. Least lignin deposition observed in unchallenged control plants emphasizes role of the biocontrol agent and MeJA in strengthening the physical defense response in wheat leaf toward the pathogen (Figure 8B).

### Effect of Biocontrol Agent and MeJA Application on Spore Germination and Disease Development

**FIGURE 9:**
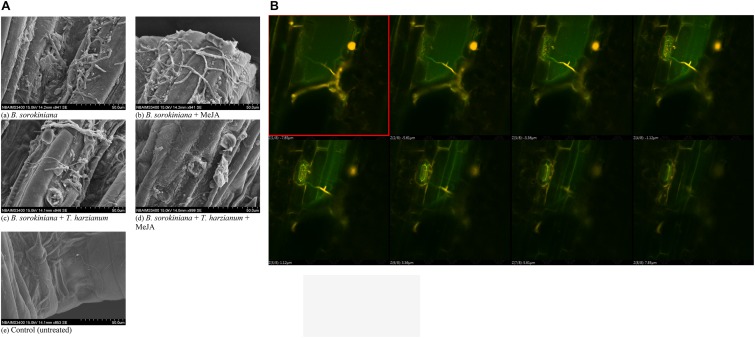
Effects of *T. harzianum* and MeJA application on **(A)** pathogen colonization under SEM study, and **(B)** pathogen colonization under confocal laser microscopic study in the plant leaves at 30 dapi, treatments were: 1 – *B. sorokiniana* (alone), 2 – *B. sorokiniana* + methyl-jasmonate (MeJA), 3 – *B. sorokiniana* + *T. harzianum* UBSTH-501, 4 – *B. sorokiniana* + *T. harzianum* UBSTH-501 + MeJA, and 5 – control (untreated). Data are mean (*n* = 5) and vertical bar lines represent standard error of mean (SEM).

Scanning electron microscopy was done to observe the leaf colonization by the *B. sorokiniana* UBS-101. Microphotographs clearly showed that the application of biocontrol agent and MeJA alone and in combination significantly reduced the colonization and proliferation of the *B. sorokiniana* UBS-101 on the plant leaves compared to other treatments (Figure 9A). Further, confocal scanning laser microscopic image clearly showed the entry of pathogen through stomata and its further proliferation (Figure 9B).

**FIGURE 10:**
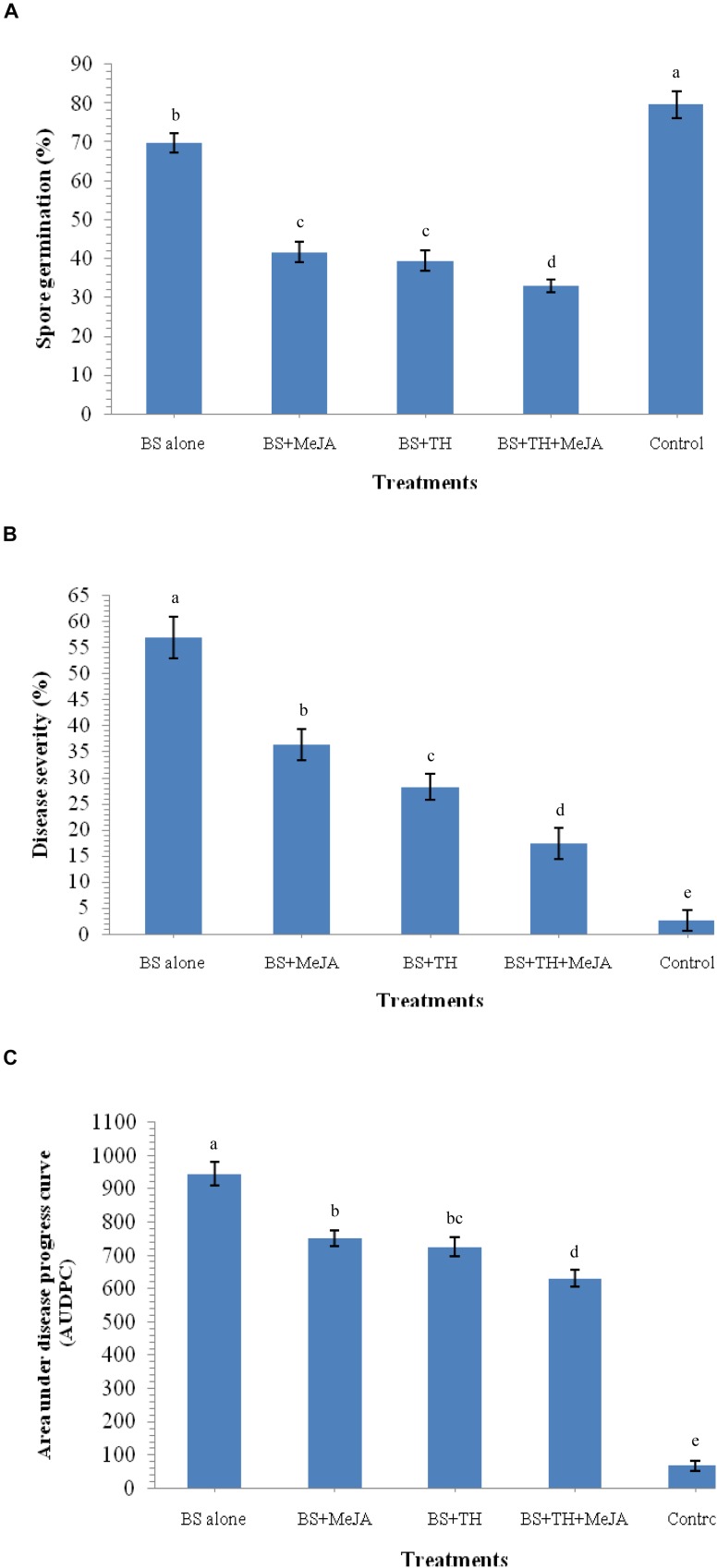
Effects of *T. harzianum* and MeJA application on spore germination and disease development after 30 DAPI, treatments, **(A)** leaf extract on spore germination after 36 h, **(B)** percent disease severity, and **(C)** AUDPC in wheat at 30 dapi, treatments were: 1 – *B. sorokiniana* (alone), 2 – *B. sorokiniana* + methyl-jasmonate (MeJA), 3 – *B. sorokiniana* + *T. harzianum* UBSTH-501, 4 – *B. sorokiniana* + *T. harzianum* UBSTH-501 + MeJA, and 5 –control (untreated). Data are mean (*n* = 5) and vertical bar lines represent standard error of mean, data with different letters show significant difference in column data in randomized block design test at *p* < 0.05 under Duncan’s multiple range test.

The reaction of fungus *B. sorokiniana* UBS-101 toward crude leaf extract was also determined. Results revealed that the leaf extract of plants pre-treated with biocontrol agents and MeJA have great potential to inhibit spore germination. A huge 69.72% spore germination of *B. sorokiniana* UBS-101 was recorded in the crude extract of plant leaves treated with *B. sorokiniana* UBS-101 only, while a still bigger figure of 79.62% was recorded in the negative control (untreated). The minimum spore germination (32.96%) recorded in the crude extract of plants pre-treated with biocontrol agent and MeJA after 36 h (Figure 10A) speaks of the effectivity of the treatment in inhibiting spore germination of the test phytopathogen.

Effect of biocontrol agent and MeJA application on disease severity and AUDPC was recorded in plants treated with *T. harzianum* UBSTH-501 and MeJA individually and also in combination (Figures 10B,C). The highest reduction in the disease severity (17.46%) and AUDPC (630.32) was recorded in the plants under pathogenic stress treated with the bioinoculant and MeJA in combination followed by plants treated with *T. harzianum* alone (28.29% and 725.45, respectively) and MeJA alone (36.47% and 751.25, respectively) under pathogenic stress conditions at 30 dapi. All the reductions in both of these parameters were statistically significant underlining the role of the treatments in reduction of the disease on practical grounds. However, highest disease severity (56.92%) and AUDPC (945.50) was recorded in plants treated with the test pathogen alone at 30 dapi (Figures 10B,C).

## Discussion

Wheat is perhaps the most important crop with global cultivation and like all other crops, suffers from a number of abiotic and biotic stresses. The fungal pathogen *Bipolaris sorokiniana* causing spot blotch of wheat is one of the most important biotic stresses of wheat leading to severe economic losses year after year over a wide geographical area. Interactions between resistant host and pathogen may get altered due to the increasing global temperature and environmental factors resulting in evolution of new races of the pathogen which are usually better equipped with for invasion of the host (Bashyal et al., 2010; Singh et al., 2016a). In the changing climatic scenario and constant increase in the area under intensive cropping, the pathogen, *B. sorokiniana* causes significant damages to wheat crop (Joshi et al., 2007). It is imperative to develop an efficient and eco-friendly alternative which can be used to control spot blotch disease. In view of the non-target hazardous effects of plant protection chemicals many researchers have advocated non-chemical methods for controlling plant pathogens including *B. sorokiniana* (Vleeshouwers and Oliver, 2014; Singh et al., 2016a). Singh et al. (2016a) had evaluated the biocontrol agents, *T. harzianum* and *Bacillus amyloliquefaciens* for control of spot blotch disease and found both the biocontrol agents effective in controlling *B. sorokiniana* in wheat crop. *Trichoderma* species are well known biocontrol agents and have been used successfully in management of a number of soil-and seed-borne phytopathogens including fungi (Gajera and Vakharia, 2012; Saravanakumar et al., 2016; Singh et al., 2016a,b), bacteria (Konappa et al., 2018; Yendyo et al., 2018), and even invertebrates (Singh et al., 2017; van Lenteren et al., 2018). As a biocontrol agent, it has potential to colonize and spread in the root, soil and foliar environments without causing any harm to the host plant and simultaneously capable of suppressing phytopathogens effectively (Sahebani and Hadavi, 2008; Singh et al., 2016a,b). Similarly, methyl jasmonate (MeJA), a major derivative of the plant hormone JA, is known to have significant effects on the activities of phytopathogens and for further elicitation of induced systemic resistance/tolerance in plants under stress conditions (Król et al., 2015). Compared with the relative wealth of information on the MeJA and its effects on plant defense in experimentally trackable plant species such as *Arabidopsis*, our understanding of the biochemical and molecular mechanisms underlying ISR in the economically important wheat crop is still in the beginning stage. The present study revealed the co-operative interaction of root-associated mutualistic plant symbiont, *T. harzianum* with methyl jasmonate for induced systemic resistance against hemi-biotroph, *B. sorokiniana* through enhanced phenylpropanoid activities in wheat. In this work, we have focused on the fungal determinants and host defense responses underlying *T. harzianum*- and MeJA-activated ISR in wheat plants challenged with *B. sorokiniana*. The results of this investigation clearly showed the efficacy of *T. harzianum* UBSTH-501 alone and in combination of MeJA in controlling *B. sorokiniana* and promoting plant growth directly and/or indirectly. Results also showed that different concentrations of MeJA reduce spore germination of *B. sorokiniana* UBS-101 significantly under *in vitro* conditions (Figure 1). This clearly indicated that MeJA has antimicrobial potential and exogenous application of MeJA may not only affect spore germination but may also directly suppress the invasion of wheat leaf tissue by the fungal pathogen under investigation. Kepczynska and Król (2012) reported that MeJA significantly inhibited spore germination and mycelial growth of *Alternaria porri* f.sp. *solani* isolated from tomato seeds *cv.* Beta. Similar reports also showed that of MeJA in different concentrations significantly inhibits the spore germination and mycelial development of other phytopathogenic fungi (Darras et al., 2005; Kepczynska and Kepczynski, 2005; Yao and Tian, 2005; Svecova et al., 2013). MeJA application increased the root colonization of wheat by *T. harzianum* significantly. One of the most important outcomes of the investigation is that upon combination, MeJA had additive effect on *T. harzianum* as a biocontrol agent. *T. harzianum* is highly diverse not only due to its wider adaptability but also the metabolites it produces. Researchers have earlier reported that a range of secondary metabolites produced by *T. harzianum* in the rhizosphere have shown inhibitory effects on the stresses including both biotic and abiotic ones depending upon the species or sometimes, even strains of a given species as well as on environmental conditions (van Wees et al., 2008; Yang et al., 2009; Vargas et al., 2011; Zachow et al., 2014).

It was also observed that plants co-treated with *T. harzianum* UBSTH-501 and MeJA produced significantly higher root biomass as compared to the uninoculated control plants (Data not shown). Apart from this, *T. harzianum* is known to increase nutrient mineralization/solubilization and mobilization in the rhizosphere which ultimately leads to a proportional increase in the uptake of nutrients, translocation of minerals and water in the plant system (Altomare et al., 1999; Harman, 2006; Arnall et al., 2009; Bae et al., 2009; Harman, 2011) contributing to improved plant health and vigor and conferring resistance to stresses thereby. These results are in agreement with the findings of Harman et al. (2004) and Singh A. et al. (2013), who demonstrated that when plants are subjected to the colonization by *T. harzianum*, and other plant growth promoting microorganisms, different cascades related to plant hormones and enzymes are up-regulated over a time period and include those probably involved in the plant growth promotion directly or indirectly (Ahn et al., 2007; Alabouvette et al., 2009; Sharma et al., 2012; Bakker et al., 2013; Ramesh et al., 2014; Alori et al., 2017). We showed that colonization of the wheat roots by the well-characterized biocontrol agent *T. harzianum* UBSTH-501 renders foliar tissues more resistant to spot blotch disease, caused by the *B. sorokiniana* UBS-101 (Figures 6, 10). Our data clearly reveal that *T. harzianum*-MeJA mediated ISR is based on direct activation of basal resistance mechanisms through enhanced activation of phenylpropanoid pathways and pronounced multifaceted cellular defense program (Vleesschauwer et al., 2008). Harman et al. (2004) reported that the activities of defense-related enzymes in plant leaves are directly related to the resistance response of the plant to the biotic stress. It has also been reported that several families of proteins from plants are associated with the regulation of the reactive oxygen species at the cellular level. Among them, superoxide dismutase, catalase; ascorbate peroxidase and peroxidase are most important which can reduce reactive oxygen species more efficiently (Lorito et al., 2010; Nanda et al., 2010; Singh et al., 2011). Manifold increased in the activities of defense-related enzymes were recorded in plants treated with MeJA and *T. harzianum* in combination compared to plants treated with either of these two individually (Figure 4). Many workers showed that SA or JA, together with ethylene and other signaling compounds activate the pathways involved in the ISR in many crops. Results showed that application of *T. harzianum* along with MeJA significantly increased the accumulation of SA and phenolic compounds, *viz.* caffeic acid, ferulic acid, *p*- coumaric acid, and chlorogenic acid in wheat plants at 0, 12, 24, 36, 48, and 60 h after pathogen inoculation (Figure 5). Further, the increased accumulation of SA and individual phenolics appear closely related to the induction of phenylpropanoid networks and induced systemic resistance in wheat. An elevated level of SA in the wheat after exogenous application of MeJA and *T. harzianum* indicated novel insight into the mechanisms and cross-talk among various networks in mitigating oxidative stress and simultaneously restricting pathogen development in wheat. It is well established that jasmonate has a central role in the regulation of the biosynthesis of several secondary metabolites in plants, including phenolic compounds, flavonoids, terpenoids, and alkaloids (Wasternack and Hause, 2013; Król et al., 2015). Researchers have reported that production of phenolic compounds, PAL and peroxidase activity may serve as markers of induced resistance to fungal diseases (Ferrer et al., 2008; Król et al., 2015). Reports also showed that jasmonates significantly affect the phenolics content in many crops (Wang and Zheng, 2005; Heredia and Cisneros-Zevallos, 2009). In the present study, results revealed that plants treated with *T. harzianum* UBSTH-501 and MeJA showed significantly higher accumulation of antioxidants and defense-related mediator molecules/enzymes in them leading to increased cell wall lignification and reduction in the rate of disease development. Moreover, *T. harzianum* and MeJA act in a cooperative manner and it was found that application of *T. harzianum* along with MeJA significantly increased lignin content and decreased disease severity and AUDPC in wheat. Scanning electron microphotographs clearly showed significantly reduced cell wall disruption and tissues disintegration by application of biocontrol agent and MeJA along with enhanced cell suberization which, in turn, restrict the invasion by pathogen. Similar effects were also observed in terms of lignin content. Results also revealed that *T. harzianum* along with MeJA elicited phenylpropanoid pathway known for imparting host resistance in the plant by reprogramming the mechanisms and cascades in wheat involving various defense-related activities like cell wall lignification, callose deposition, accumulation of phytoalexins, and other metabolites toxic to the pathogens. Lignification increases in response to biotic stress and represents adoptive mechanisms to restrict the entry of pathogens due to the antimicrobial and non-degradable traits of lignin (Dong, 2004; Dong et al., 2004; Harman et al., 2004; Dodds and Rathjen, 2010; Sarma et al., 2015). In brief, the present study describes overall mechanisms involved in the *T. harzianum*- and MeJA-mediated disease control in *B. sorokiniana*-wheat pathosystem. The application of *T. harzianum* and MeJA revealed well-coordinated modes of action during pathogen attack, expression of defense-related mediator molecules/enzymes and cell wall lignification ultimately leading to the reduction in the rate of pathogenic colonization, disease development and improved plant growth under biotic stress condition.

## Conclusion

It has been observed that combined application of *T. harzianum* UBSTH-501 and MeJA significantly increased the activation and accumulation of defense-related biomolecules in wheat plants even after a few hours of pathogen inoculation under pathogenic stress conditions. MeJA could be a very promising compound for the activation of phenylpropanoid pathways under pathogenic stress at an early stage which is further maintained by *T. harzianum* in a cooperative manner. It is clear that MeJA is part of an extremely complex signal transduction network. It may either directly influence the activities of certain defense-related enzymes, as described for PAL, peroxidase, catalase, or SOD, or may directly or indirectly induced gene responsible for protective mechanisms in wheat plants under biotic stress. Furthermore, when wheat plants were inoculated with both the agents *T. harzianum* UBSTH-501 and MeJA, a cooperative interaction was recorded. It was also noticed that when plants are treated with *T. harzianum* UBSTH-501 and MeJA, the synthesis of SA in the plant tissue is significantly increased which may further activate cascades related to SAR and save the plants from fungal infection. It was also found that application of *T. harzianum* UBSTH-501 and MeJA alone or in combination significantly suppresses disease development and reduces disease severity and AUDPC. Their application also increased lignin content in plant leaves which further reduces the infection, colonization and invasion process by the pathogen. With the help of these findings, we conclude that *T. harzianum* UBSTH-501 along with MeJA (150 μg ml^–1^) could be a potential alternative of toxic chemical fungicides and can be applied at larger scale to control spot blotch disease in wheat at experimental plots and farmers’ field.

## Author Contributions

US, DM, SS, MI, JR, ASh, and ASa were involved in conceiving the idea and designing the experiments. US, DM, SS, MK, MR, and PS conducted the research work. PS and MR contributed to the CSLM and SEM studies, respectively. US, DM, HS, JR, PS, and SK analyzed the data and prepared the manuscript. All authors contributed to the interpretation of results and discussion. All authors have approved the final version of the manuscript.

## Conflict of Interest Statement

The authors declare that the research was conducted in the absence of any commercial or financial relationships that could be construed as a potential conflict of interest.
